# On a Two-Phase Size-Structured Population Model with Infinite States-At-Birth and Distributed Delay in Birth Process

**DOI:** 10.1080/17513758.2014.899637

**Published:** 2014-03-25

**Authors:** Meng Bai, Shihe Xu

**Affiliations:** School of Mathematics and Statistics, Zhaoqing University, Zhaoqing, Guangdong 526061, People's Republic of China

**Keywords:** size-structured populations, distributed delay, well-posedness, asynchronous exponential growth, semigroups

## Abstract

In this paper we study the two-phase size-structured population model with infinite states-at-birth and distributed delay in birth process. The model distinguishes individuals by two different status: the ‘reproductive’ stage and the ‘nonreproductive’ stage. We establish the well-posedness for this model and show that the solution of this model exhibits asynchronous exponential growth by means of semigroups. We also consider a special case in which the individuals in the ‘reproductive’ stage and the ‘nonreproductive’ stage have the same growth rates and give a comparison between this two-phase model with the classical one-phase model.

## 1. Introduction

Among those size-structured population models with infinite states-at-birth, the fundamental one is as follows:

(1)
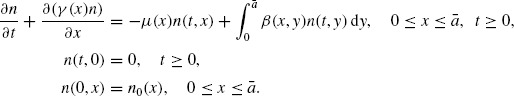


Here, the unknown function *n*(*t, x*) denotes the density of individuals of size 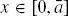 at time *t* ∊ [0, ∞), where ā > 0 represents the (finite) maximum size of any individual in the population. The vital rates μ(*x*) and γ(*x*) denote the death and growth rates, respectively. It is assumed that individuals may have different sizes at birth and therefore β(*x, y*) denotes the probability which individuals of size *y* give birth to the individuals of size *x.* The asymptotic behaviour of its solution can be obtained by using the generalized relative entropy method [12]. Recently the similar nonlinear model was studied in [10,11]. An interesting situation to consider is when the birth process undergoes an observable period so that there is a time lag between conception and birth [6,15,16,18]. In such considerations, the model (1) should be improved by the following model with the distributed delay:

(2)
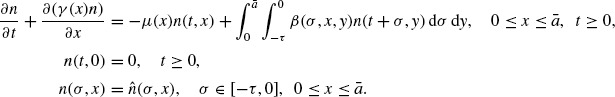


Here β(σ, *x, y*) denotes the rate at which individuals of size *y* give birth to the individuals of size *x* after a time lag -σ starting from conception and τ is a constant denoting the maximal delay. For example, in a host-parasite system, the distributed delay in model (2) may be given by the time lag between laying and hatching of the parasite eggs [2]. Moreover, unlike the non-distributed delay case, the time lag considered here can change from 0 to τ, i.e. it is distributed in the interval [0, τ]. The idea of considering the distributed delay is inspired by the work of Piazzer and Tonetto [14], where a different age-structured population model with distributed delayed birth process was studied. Recently, the model (2) has been proved in [4] that the problem is globally well-posed, and the solutions exhibit so-called asynchronous exponential growth (we refer the readers to [1,4,9,14] for definition).

In this paper we study a model which distinguishes individuals by two different status: the ‘reproductive’ stage and the ‘nonreproductive’ stage. In this model, only individuals in the ‘reproductive’ stage reproduce. In fact the ‘reproductive’ stage and the ‘nonreproductive’ stage occur in the evolution of many populations generally. We denote *by p*(*t, x*) and *n*(*t, x*) the densities of individuals in the ‘reproductive’ stage and the ‘nonreproductive’ stage of size *x* ∊ [0, ¯] at time *t* ∊ [0, ∞), respectively. Then the model reads as follows:

(3)
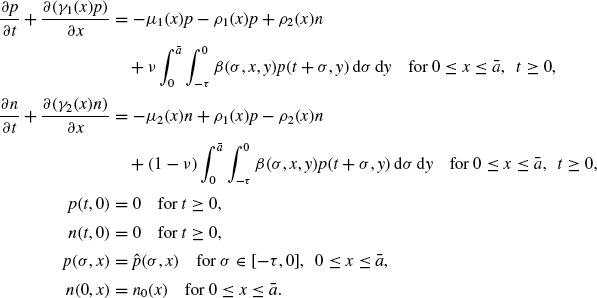


Here γ_1_ (*x*) and γ_2_ (*x*) represent the growth rates of the individuals in the ‘reproductive’ stage and in the ‘nonreproductive’ stage, 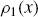 and ρ_2_ (*x*) represent the transferring rates between the ‘reproductive’ stage and the ‘nonreproductive’ stage, respectively, and μ_1_(*x*) and μ_2_(*x*) represent the death rates of the individuals in the ‘reproductive’ stage and the ‘nonreproductive’ stage, respectively. Also β(σ, *x, y*) represent the rate at which the individuals in the ‘reproductive’ stage of size *y* give birth to the individuals in the ‘reproductive’ stage or the ‘non-reproductive’ stage of size *x* after a time lag – σ starting from conception, and *v* is a constant, 0 ≤ *v* ≤ 1. In addition, 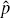 and *n*_0_ are given functions defined in [-τ, 0] × [0, ā] and [0, ā], respectively. Later on we shall denote

(4)
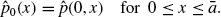


The similar model without distributed delay has been proved in [9] that the problem is globally well-posed, and the solutions exhibit asynchronous exponential growth. The purpose of this work is to extend the results in [4,9] to the model. We shall prove that under suitable assumptions on μ_1_(*x*), μ_2_(*x*), ρ_1_(*x*), ρ_2_(*x*), γ_1_(*x*), γ_2_(*x*), β(σ, *x, y*) and (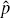, *n*_0_) the model (3) is globally well-posed, and its solution possesses the properties of asynchronous exponential growth.

Throughout this paper, μ_1_(*x*), μ_2_(*x*), ρ_1_(*x*), ρ_2_(*x*), γ_1_(*x*), γ_2_(*x*) and β(σ, *x, y*) are supposed to satisfy the following conditions:

(H.1) μ_1_, μ_2_, ρ_1_ and ρ_2_ are nonnegative and continuous functions defined on [0, ā].
(H.2) γ_1_, γ_2_ ∊ *C*^1^ [0, ā] and γ_1_ (*x*), γ_2_(*x*) > 0 for all *x* ∊ [0, ā].
(H.3) 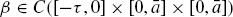 and β ≥ 0.

In order to prove the property of asynchronous exponential growth, we make the additional assumptions:

(H.4) If 0 ≤ *v* < 1, ρ_2_(*x*) > 0 for all *x* ∊ [0, ā]. If *v* = 1, ρ_1_(*x*), ρ_2_(*x*) > 0 for all *x* ∊ [0, ā].
(H.5) β(., *x, y*) > 0 for *y > x*.

We introduce the subspace 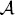 of 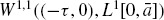 and the subspace ℬ of *W*^1,1^(0, ā) as follows:


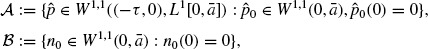


where 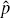
_0_ is defined by Equation (4). Note that since 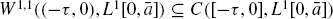, for any 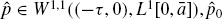 is well defined. Similarly, since 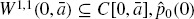 and *n*_0_ (0) are also well defined.

Our first main result establishes the global well-posedness of the problem (1) and reads as follows:

Theorem 1.1 For any 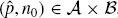, the model (3) has a unique solution 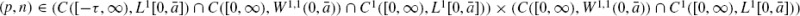. Moreover, for any *T* > 0, the mapping 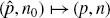 from 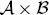 to 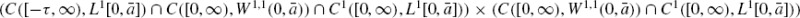 is continuous.

The proof of this result will be given in Section 2.

Actually, from the proof of Theorem 1.1 we shall see that for any *F* ∊ ε, where


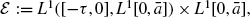


the model (3) has a unique so-called mild solution (*p, n*) in the sense that 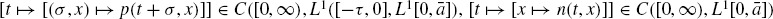 and it satisfies an integral equation which is equivalent to Equation (3) in a suitable sense. This defines, for each *t* ≥ 0, an operator 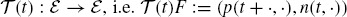. Later, we shall see that 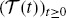 is a strongly continuous semigroup in ε.

Our second main result of this article studies the asymptotic behaviour of this semigroup and reads as follows:

Theorem 1.2 There exist a rank one projection Π on ε; and constants 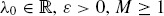 such that
(5)
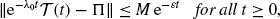

where || · || denotes the operator norm on ε.

The proof of this result is given in Section 3. The parameter λ_0_ is called *intrinsic rate of natural increase* or *Malthusian parameter* [17]. Theorem 1.2 shows that the solutions of the model (3) exhibit asynchronous exponential growth.

Next we consider the special case of the model (3), where γ_1_(*x*) = γ_2_(*x*) = γ (*x*) and give a comparison between this two-phase model with the one-phase model. We want to give a comparison between the asymptotic behaviours of the sum of the densities of individuals in the ‘reproductive’ stage and the ‘nonreproductive’ stage and the solution of the one-phase model after modifying the death rates and β(σ, *c, y*) properly. Note that the above result says that there exists a positive vector function 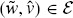, such that for any 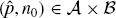, the mild solution (*p*(*t + .,.*), *n*(*t, .*)) of the model has the following asymptotic expression:


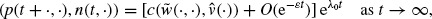


where *c* is a constant uniquely determined by the initial data 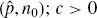 provided 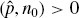 (i.e. 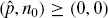 and 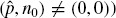). Wedenote


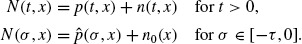


We want to compare *N*(*t, x*) with the solution 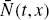 of the problem

(6)
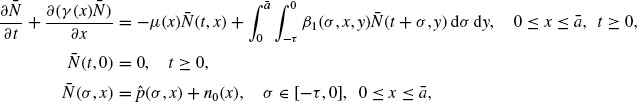


where


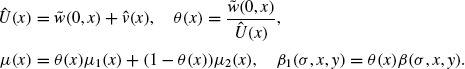


We can see that θ(*x*) is the asymptotic proportion of the individuals in the ‘reproductive’ stage in the population. Since the model (6) describes the evolution of the sum of the densities of individuals in the ‘reproductive’ stage and the ‘nonreproductive’ stage in the asymptotic sense, one might expect that 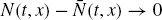 as *t → ∞*. But to our surprise, this is actually not the case. In fact, we have the following result:

Theorem 1.3 Let the notation be as above. We have the following relation:

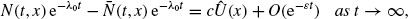

where *c* is a constant which is generally non-vanishing.

The proof of this result will be given in Section 3. Theorem 1.3 shows that the asymptotic behaviours of the sum of the densities of individuals in the ‘reproductive’ stage and the ‘nonre-productive’ stage and the solution of the one-phase model are different and the research of the model with two stages is meaningful.

The layout of the rest of the paper is as follows. In Section 2 we reduce the model (3) into an abstract Cauchy problem and establish the well-posedness of it by means of strongly continuous semigroups. In Section 3, we prove that the solution of the model (3) has asynchronous exponential growth. In Section 4, we give the proof of Theorem 1.3.

## 2. Reduction and well-posedness

In this section we reduce the problem (1) into an abstract Cauchy problem and establish the well-posedness of it by means of strongly continuous semigroups. We refer the reader to see [5,14] for similar reductions.

First, we introduce the following operators on the Banach spaces 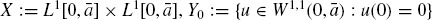 and 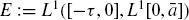:


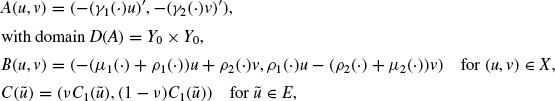


where


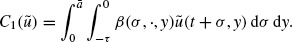


We note that 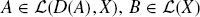 and 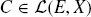. Using this notation, we rewrite the model (1) into the following abstract initial value problem for a retarded differential equation in the Banach space *X*:

(7)
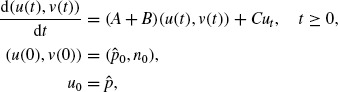


where 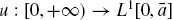 and 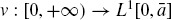 are defined as *u*(*t*): = *p*(*t,.*) and *v*(*t*) := *n*(*t,.*), respectively, and 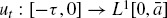 is defined as *u_t_*(σ) := *p*(*t + σ*), σ ∊ [-τ, 0].

Remark 2.1 As usual, if the functions 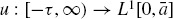 and 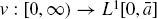 satisfy 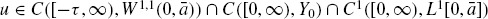 and 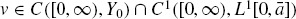, respectively, and the problem (7) in usual sense, we say that functions (*u, v*) is a *classical solution* of the problem (7). It is evident that a necessary condition for the problem (7) to have a classical solution is that 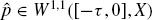 and the functions 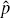
_0_ defined by Equation (4) and *n*_0_ belong to *Y*_0_.

Next, we introduce the following operators in the Banach space *E*:


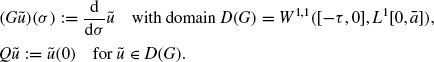


We note that 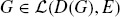 and 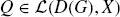. We now let 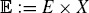, and introduce operators **A**_0_, **B** and **A** in E as follows:


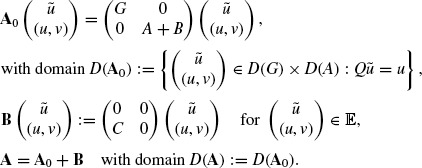


We note that 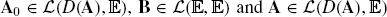. Using this notation, we see that the problem (7) can be equivalently rewritten into the following abstract initial value problem of an ordinary differential equation in the Banach space E:

(8)
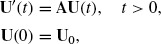


where


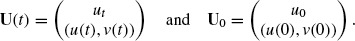

Remark 2.2 As usual, we say that a function 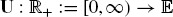 is a *classical solution* of the problem (8) if 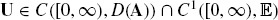 and if it satisfies Equation (8) in the usual sense.

To be rigorous, we write down the following preliminary result:

Lemma 2.1 Let the necessary condition mentioned in Remark 2.2 is satisfied. If 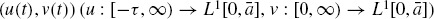 is a classical solution of the problem (7), then 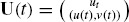 is a classical solution of the problem (8). Conversely, if ***U*** is a classical solution of the problem (8), then **U** has the form 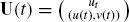 for all *t* ≥ 0, and by extending the first component of its second component 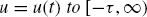 such that *u*(*t*) = *u*_0_ for *t* ∊ [–τ, 0), we have that (*u, v*) is a classical solution of the problem (7).

Proof See Lemma 2.1 of Bai and Xu [4] and Theorem 2.2 of Piazzer and Tonetto [14].

In the sequel, we consider the semigroup generated by the operator **A**. We first consider the one generated by the principle part **A**_0_ of **A**. We have the following results:

Lemma 2.2 The component operator *A* + *B* of operator matrix **A**_0_ generates a strongly continuous semigroup 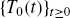 on *X*.

Proof We note that *A* generates a nilpotent semigroup on *X*(see Theorem 2.2 of Farkas and Hinow [9]). Since *B* ∊ *L*(*X*), by using the perturbation theorem for generators of strongly continuous semigroups in Banach spaces (see Theorem III.1.3 of Engel and Nagel [8]), we get this lemma.

Lemma 2.3 The operator **A**_0_ generates a strongly continuous semigroup 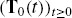 on 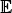, given by
(9)
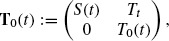

where (*S*(*t*))_*t*≥0_ is a nilpotent left shift semigroup on E, given by
(10)
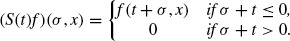

and *T*_*t*_: *X* → *E* are linear operators defined as
(11)
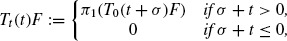

where π_1_ is the projection onto the first coordinate.

Proof See [5, Theorem 2.2] and Lemma IV.1.2 of Engel and Nagel [8].

Since 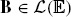, by using the perturbation theorem for generators of strongly continuous semigroups in Banach spaces (see [8, Theorem III.1.3]), we get the following lemma:

Lemma 2.4 The operator ***A*** generates a strongly continuous semigroup 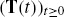 on *E*.

By using the theory of strongly continuous semigroups in Banach spaces, we get the following result:

Theorem 2.5 For any given initial data 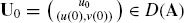, the problem (8) has a unique solution 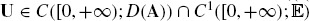, given by
(12)
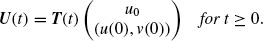

By Lemma 2.1 and Theorem 2.5, we see that Theorem 1.1 follows.

## 3. Asynchronous exponential growth

In this section we study the asymptotic behaviour of the solution of the problem (1). We shall prove that the semigroup (**T**(*t*))_*t*≥0_ has the property of asynchronous exponential growth on E. We denote by ω_ess_(*A*) the *essential growth bound* of the semigroup (*T*(*t*))_*t*≥0_ with generator *A,*

i.e.

(13)
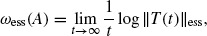


ω_0_(*A*) the *growth bound*, i.e.

(14)
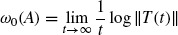


and *s*(**L**) the *spectral bound*, i.e.


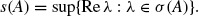


If we prove that the semigroup (**T**(*t*))_*t≥*;0_ is an irreducible positive strongly continuous semigroup (we refer the readers to [7,8] for definition) satisfying the inequality ω_ess_(**A**) < ω_0_(**A**), then by [7, Theorem 9.10 and Theorem 9.11], the semigroup (**T**(*t*))_*t*≥0_ has the property of asynchronous exponential growth on E. Thus, in the sequel we step-by-step prove the above assertions about the semigroup (**T**(*t*))_*t*≥0_.

Lemma 3.1 The semigroup (**T**(*t*))_*t*≥0_ generated by **A** is positive and eventually compact (we refer the readers to [7,8] for definition).

Proof Since **B** is a positive bounded linear operator in E, the positivity of (**T**(*t*))_*t*≥0_ follows if we prove that the semigroup (**T**_0_ (*t*))_*t*≥0_ generated by **A**_0_ is positive (see Corollary VI.1.11 of Engel and Nagel [8]). Since (*S*(*t*))_*t*≥0_ is positive, by the expression (9) of (**T**_0_(*t*))_*t*≥0_, we only need to prove that the semigroup (*T*_0_(*t*))_*t*≥0_ generated by *A + B* is positive. The positivity of *A + B* can be easily obtained by the characteristic method (see [3, Lemma 2.1] and [9, Theorem 2.2]). Therefore the positivity of (**T**(*t*))_*t*≥0_ follows. From the proof of [9, Lemma 3.6], we have that *T*_0_ (*t*) = 0 for 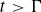, where 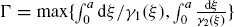. From Equation (1), we also see that *T_t_*(*t*) = 0 for *t* > Γ + τ. Hence, from Equations (9) and (10), we have that **T**_0_(*t*) = 0 for *t* > Γ + τ. This particularly implies that (**T**_0_(*t*))_*t*≥0_ is compact for *t* > Γ + τ. Thus, by Proposition III.1.14 of Engel and Nagel [8], the eventual compactness of (**T**(*t*))_*t*≥0_ follows if we prove that **B** is compact. We note that the only nonzero component operator of operator matrix **B** is *C: E → X.* We use the method which is similar to Lemma 3.6 in [9] to prove that *C* is compact. Hence the desired assertion follows.

By the eventual compactness of the semigroup (**T**(*t*))_*t*≥0_ and Corollary IV.3.12 of Engel and Nagel [8], the following result holds.

Lemma 3.2 *s*(***A***) = ω_0_(***A***).

In the proof of Lemma 3.1, we have that the semigroup **T**_0_(*t*) = 0 for *t* > Γ + τ. Then by the definition (14), we have that ω_ess_(**A**_0_) = -∞. Since **B** is compact on E, by Proposition 2.12 of Clément *et al.* [7], we have the following result:

Lemma 3.3 ω_ess_(***A***) = -∞.

Lemma 3.4 The semigroup (**T**(*t*))_*t*≥0_ generated by **A** is irreducible.

Proof By Lemma VI.1.9 of Engel and Nagel [8] and Lemma 3.4 of Bai and Cui [3], if we prove that (*R*(λ, **A**)**F**, Ψ) > 0 for some λ > 0 and all **F** ∊ E and 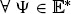 such that **F** > 0 and Ψ > 0, then the desired assertion follows. Let *π*_1_ and *π*_2_ be the projections onto the first and the second coordinates, respectively. We will prove that *π*_1_(*R*(λ,**A**)**F**)(σ, *x*) > 0 for almost all 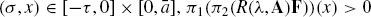 and 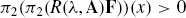 for almost all 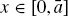. To this end, we first deduce an useful expression of *R*(λ, **A**). For 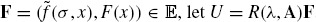. Then **U** satisfies the equation
(15)
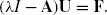

By writing 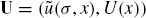 and 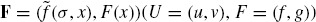, we see that the above equation can be rewritten as follows:
(16)
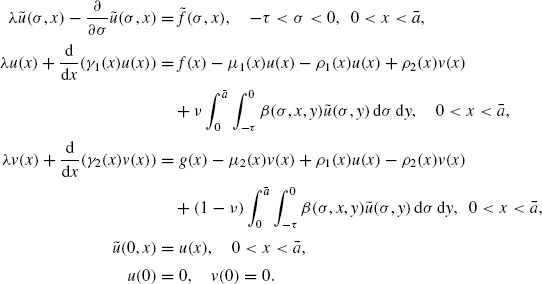

Then

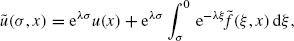

where 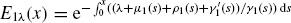 and 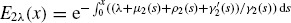.

For each 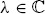, we define two operators *M_λ_* and *N*_λ_ on **E** as follows:
(17)
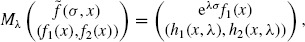

(18)
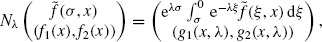

where

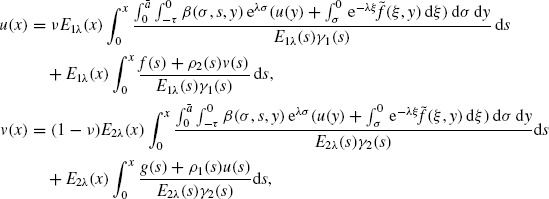

Since
(19)
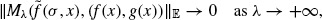

there exists *λ*∗ > 0 such that ||*M_λ_*|| < 1 for *λ ≥ λ∗*. This implies that the inverse (*I – M_λ_*)^–1^ exists and is a bounded operator for *λ ≥ λ∗*. From Equations (17)–(19), we see that the resolvent of **A** is given by
(20)
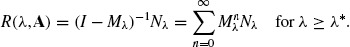

We first consider the case in which 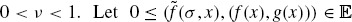 and 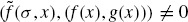. Without loss of generality, we assume that 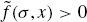 for almost all 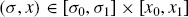 with some 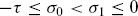 and 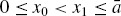. Then for almost all 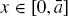,

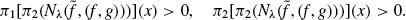

This is for almost all 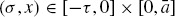,

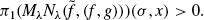

From Equation (20), we obtain that *π*_1_(*R*(λ, **A**)**F**)(σ, *x*) > 0 for almost all (σ, *x*) ∊ [τ, 0] × [0, ā] and *π*_2_(*R*(λ,**A**)**F**)(*x*) > 0 for almost all *x* ∊ [0, ā]. If we assume that *f*(*x*) > 0 for almost all *x* ∊ [*x*_0_, *x*_1_] with some 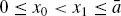. Then for almost all *x* ∊ [*x*_0_, ā],

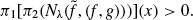

This further implies that for almost all *x* ∊ [0, ā],

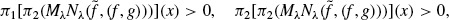

and for almost all (σ, *x*) ∊ [-τ, 0] × [0, ā],

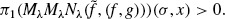

Then the result is the same. If we assume that *g*(*x*) > 0 for almost all *x* ∊ [*x*_0_, *x*_1_] with some 0 ≤ *x*_0_ < *x*_1_ ≤ ā. Then for almost all *x* ∊ [*x*_0_, ā],

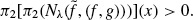

This further implies that for almost all *x* ∊ [*x*_0_, ā],

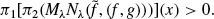

Then for almost all *x* ∊ [0, ā],

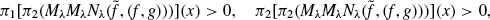

and for almost all (σ, *x*) ∊ [-τ, 0] × [0, ā],

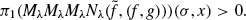

Then the result is also the same. If we assume that ν = 0 or ν = 1, the arguments are the similar by the assumption (H.4). This completes the proof.

By the positivity of the semigroup (**T**(*t*))_*t*≥0_, *s*(**A**) > -∞(see Theorem VI.1.10 of Engel and Nagel [8]), Corollary 3.2 and Lemma 3.3, we have the following result:

Lemma 3.5 *s*(**A**) ∊ σ(**A**) and ω_ess_(**A**) < *s*(**A**).

By Lemmas 3.1–3.5 and Corollary V.3.3 of Engel and Nagel [8], we conclude that there exist a positive rank one projection operator π in E and constants *ε*; > 0 and *M* ≥ 1 such that

(21)
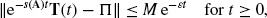


where || · || denotes the operator norm in E. This completes the proof of Theorem 1.2.

## 4. Relation with the one-phase model

In this section we give the proof of Theorem 1.3 and give a comparison with the sum of the densities of individuals in the ‘reproductive’ stage and the ‘nonreproductive’ stage *N*(*t, x*) and the solution 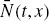 of the problem (6). The idea is inspired by the generalized relative entropy method [12,13]. In [3], we use the similar method to give a comparison between a two-phase cell division model with the classical one-phase model.

Proof of Theorem 1.3 By Lemma 3.5, we know that λ = *s*(**A**) is the dominant eigenvalue of the eigenvalue problem
(22)
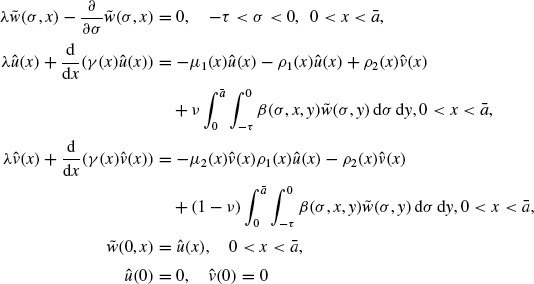

and the corresponding eigenvector 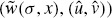 is strictly positive, i.e. 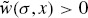 for all 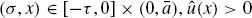 and 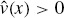 for all 0 < *x* < ā. Letting

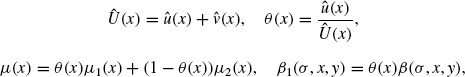

we have that
(23)
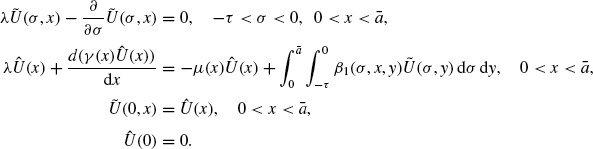

Hence, *λ = s*(**A**) is also an eigenvalue of the above eigenvalue problem, and 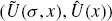 is the corresponding eigenvector. Since 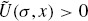 for all 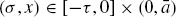 and 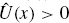 for all 0 < *x* < ā, it follows that *λ = s*(**A**) is also the dominant eigenvalue of the above eigenvalue problem.

Now let (*p*(*t,x*), *n*(*t,x*)) and 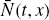 be the solutions of the model (3) and (5) with initial data 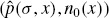 and 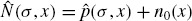, respectively, and set

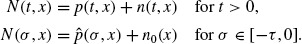

We want to compare *N*(*t, x*) and 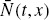. From Equation (21) and Theorem 9.11 of Clément *et al.* [7], we have the following asymptotic expression:
(24)
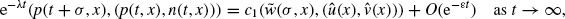

where *c*_1_ is a constant uniquely determined by the initial data 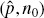; *c*_1_ provided 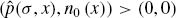 (i.e. 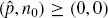 and 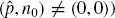. This implies that
(25)
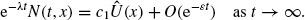

From Equation (21) of Bai and Xu [4] and Theorem 9.11 of Clément *et al.* [7], we have the following asymptotic expression:
(26)
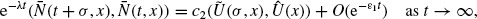

where *c*_2_ is a constant uniquely determined by the initial data 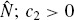 provided 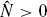 (i.e. 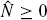 and 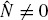). This also implies that
(27)
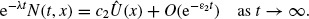

From Equations (25) and (27), we obtain
(28)
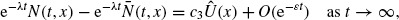

where *c*_3_ = *c*_2_ – *c*_1_ and *ε*; = min{*ε*;_1_, *ε*;_2_}. In what follows we prove that, generally speaking, *c*_3_ = 0. Let (φ, (φ, ψ)) be the eigenvector of the conjugate problem of Equation (22) (see Theorem 8.17 of Clément *et al.* [7]), i.e.
(29)
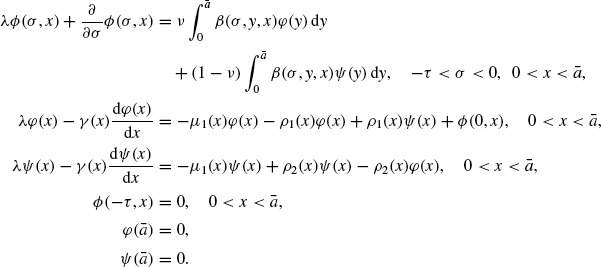

We normalize (φ, (φ, ψ)) such that
(30)
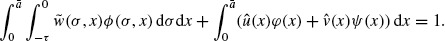

By Theorem 8.17 of Clément *et al.* [7], φ(σ, *x*) > 0 for all (σ, *x*) ∊ [τ, 0] × (0, ā), φ(*x*) > 0 and ψ(*x*) > 0 for all 0 < *x* < ā, due to a similar reason as that for 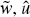 and 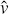. Next, let (Φ, Ψ) be the eigenvector of the conjugate problem of Equation (23) (see Theorem 8.17 of Clément *et al.* [7]), i.e.
(31)
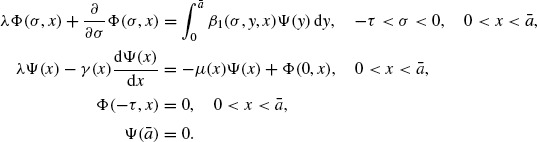

We normalize (Φ, Ψ) such that

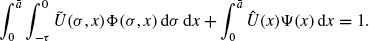

By Theorem 8.17 of Clément *et al.* [7], Φ(σ, *x*) > 0 for all 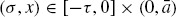 and Ψ(*x*) > 0 for all 0 < *x* < ā. From Equations (3) and (29), we easily obtain

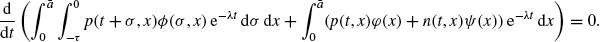

Hence

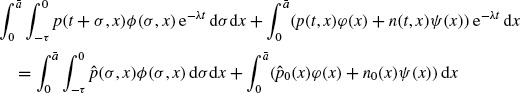

for all *t* ≥ 0. Letting *t* → ∞ and using (24), we get

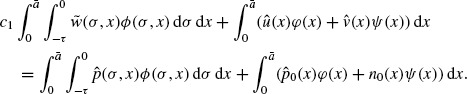

From Equation (30), we obtain

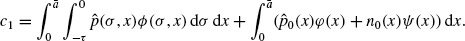

By a similar argument we have

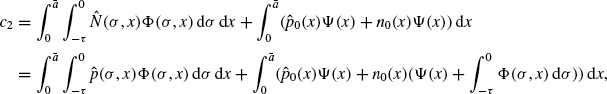

so that generally speaking we have *c*_1_ ≠ *c*_2_ or *c*_3_ ≠ 0. This proves Theorem 1.3.

## References

[1] Arina O., Bertuzzi A., Gandolfi A., Sánchez E., Sinisgalli C. (2005). The asynchronous exponential growth property in a model for kinetic heterogeneity of tumour cell populations. J. Math. Anal. Appl..

[2] Auslander D.M., Oster G.F., Huffaker C.B. (1974). Dynamics of interacting populations. J. Franklin Inst.

[3] Bai M., Cui S. (2012). Analysis of a two-phase cell division model. Appl. Math. Comput..

[4] Bai M., Xu S. ((2013)). On a size-structured population model with infinite states-at-birth and distributed delay in birth process. Appl. Anal..

[5] Bátkai A., Piazzera S. (2001). Semigroups and linear partial differential equations with delay. J. Math. Anal. Appl..

[6] Blasio G.D. (1979). Nonlinear age-dependent population growth with history-dependent birth rate. Math. Biosci..

[7] Clément Ph., Heijmans H., Angenent S., van Duijin C., de Pagter B. (1987). One-Parameter Semigroups.

[8] Engel K-J., Nagel R. (2000). One-Parameter Semigroups for Linear Evolution Equations.

[9] Farkas J.Z., Hinow P. ((2010)). On a size-structured two-phase population model with infinite states-at-birth. Positivity.

[10] Farkas Z., Hinow P. (2012). Steady states in hierarchical structured populations with distributed states at birth. Discrete Contin. Dyn. Syst. – Ser. B.

[11] Farkas J.Z., Green D.W., Hinow P. (2010). Semigroup analysis of structured parasite populations. Math. Model. Nat. Phenom..

[12] Perthame B. (2007). Transport Equations in Biology.

[13] Perthame B., Ryzhik L. (2005). Exponential decay for the fragmentation or cell-division equation. J. Diferential Equations.

[14] Piazzera S., Tonetto L. (2005). Asynchronous exponential growth for an age dependent population equation with delayed birth process. J. Evol. Equations.

[15] Swick K.E. ((1977)). A nonlinear age-dependent model of single species population dynamics. SIAM J. Appl. Math..

[16] Swick K.E. ((1980)). Periodic solutions of a nonlinear age-dependent model of single species population dynamics. SIAM J. Math. Anal..

[17] Thieme H.R. (1998). Balanced exponential growth of operator semigroups. J. Math. Anal. Appl..

[18] Zhu G.B., Chan W.L. ((1989)). A semigroup approach to age dependent population dynamics with time delay. Commun. Partial Differential Equations.

